# Management of pain in the cancer patient

**DOI:** 10.3389/fpain.2022.926712

**Published:** 2022-08-08

**Authors:** Shalini Dalal, Eduardo Bruera

**Affiliations:** Department of Palliative Care and Rehabilitation Medicine, The University of Texas MD Anderson Cancer Center, Houston, TX, United States

**Keywords:** cancer pain, opioid crisis, pain assessment, psychology and pain, palliative care, cancer pain relief

## Introduction

**Figure 1:**
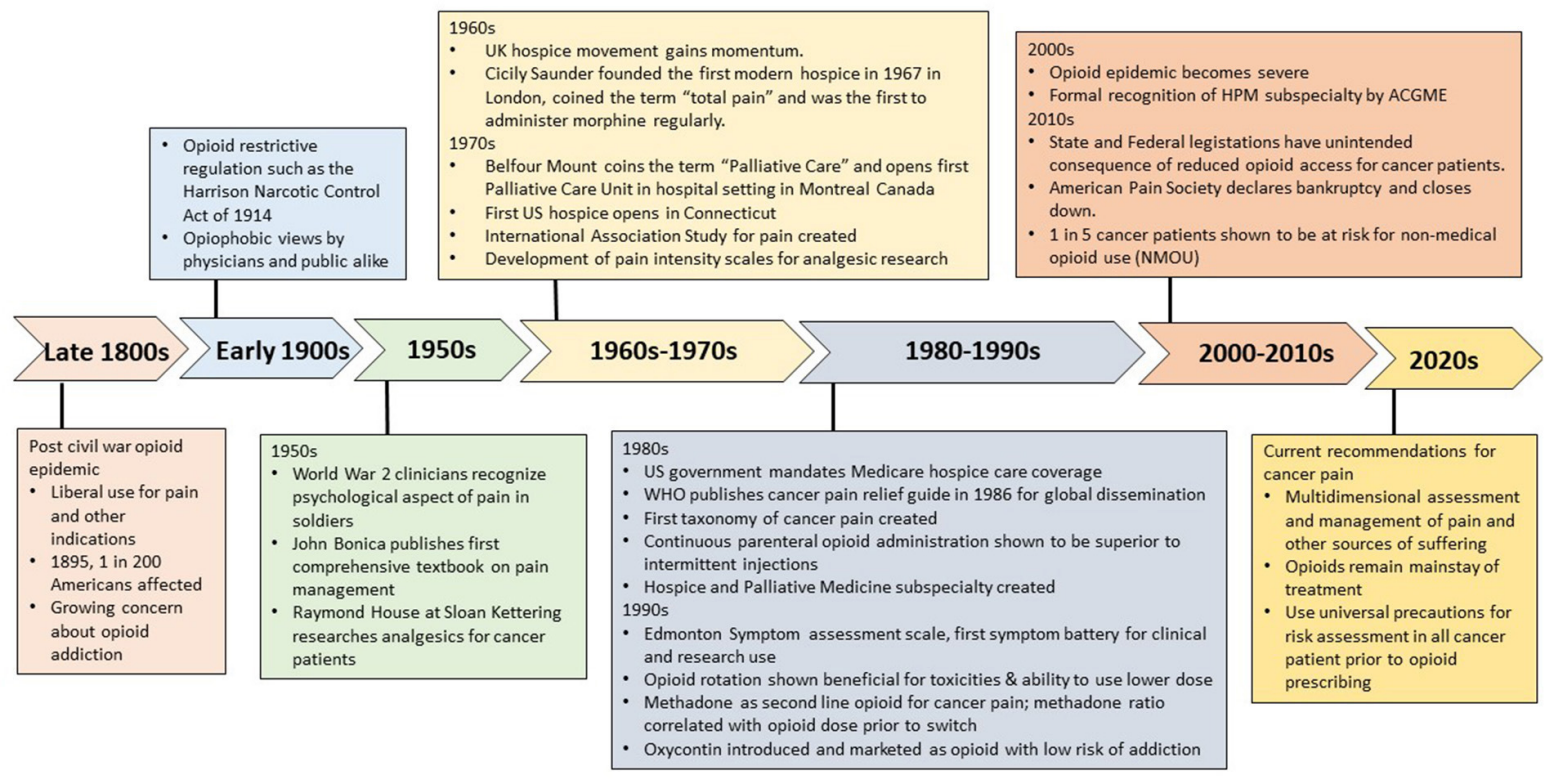
Pivotal events in cancer pain management.

Opioids are well-recognized as the mainstay of pharmacological treatment for cancer pain (1). However, concerns about their effectiveness, safety and misuse liability have evolved over time, with some decades marked by an overly restrictive stance on opioid use with reluctance to prescribe even for severe cancer pain, and other decades associated with a push for greater acceptance to endorse this treatment. Here we briefly describe historical milestones in the evolution of cancer pain management (Figure 1), focusing on the work of revolutionary clinicians who by placing great emphasis on scientific evidence and education were able to bring upon transformative changes to cancer pain relief. We will also discuss the complex issue facing us —the interface between providing adequate analgesia to cancer patients and inherent risk of non-medical opioid use (NMOU) and opioid use disorder.

## The first half of the 20^th^ century: Opiophobic views dominate

In the decades after the Civil War, the U.S. faced an epidemic of opioid addiction related to the use of morphine injections among war veterans, and by 1895 it affected ~1 in 200 Americans, (2, 3) compelling medical professionals to turn against opioids and support overly restrictive legislations such as the Harrison Narcotic Control Act of 1914 (2). For the most part, physicians' opiophobic views dominated the first half of the 20^th^ century (4), even for terminal cancer patients who at diagnosis had little hope for cure or relief from their dreaded disease (5). A crucial turning point for cancer emerged mid-century following the discovery of cytotoxic chemotherapies with prioritization of national funding for cancer research, yet patient suffering and the obvious lack of an organized approach for providing relief, had little effect on policies (6).

## Observed lessons of war: Psychology affects pain perception

During World War II, clinicians such as William Livingston, Henry Beecher and John Bonica, treating wounded soldiers made fundamental observations that the patient's pain perception and experience was influenced by individual circumstances, galvanizing research into the psychological aspects of pain (7). Around this time, Raymond Houde at Memorial Sloan-Kettering Cancer Center, studying the effects of various analgesics to treat cancer patients, (8) was one of the first to outline pain management strategies that considered the cancer patient's psychology, prognosis and preference (9). These independent events were instrumental in recognizing the need to explore psychological and social factors in evaluation of pain.

## The emergence of palliative care discipline

Across the Atlantic, in the early 1960's, the UK hospice movement was gaining momentum, providing care to marginalized terminally ill patients in facilities outside mainstream medicine (10). Here, Dr Cicely Saunders, considered the founder of the modern hospice movement, coined the term “total pain” to describe the complex interplay between physical, emotional, and existential suffering (11). In 1967, she founded St Christopher's Hospice in London, the first modern hospice that combined education and research with interdisciplinary patient care (10). Saunders' observations guided her practice of providing morphine on a regular schedule for superior pain relief as compared to the as needed approach.

In the U.S., the first hospice was founded in 1974 by Florence Wald in Connecticut (12). The same year, Balfour Mount, a surgical oncologist, coined the term “palliative care,” differentiating it from hospice care, and in 1976, opened the first inpatient palliative care unit at a university hospital in Montreal, Canada (12). This represented the first of its kind specialized unit that cared for terminally ill patients at a mainstream medical facility and was subsequently adopted by other institutions and cancer centers in North America (12, 13). In 1980, the US federal government's mandate that hospice care be covered by Medicare was crucial in its widespread adoption^1^. In 1986, Hospice and Palliative medicine specialty was created and formally recognized by the Accreditation Council for Graduate Medical Education in 2006 (14).

## Changing times: Globalization of cancer pain relief

Ronald Melzack and Patrick Wall's “gate control” theory of pain initially published in 1965 generated much interest and revolutionized pain research, (15) offering for the first time a physiological explanation for the previously observed effect of psychology on pain perception. In 1973, Bonica organized the first international pain meeting and the following year founded the International Association for the Study of Pain (IASP). Several IASP meetings specifically addressed cancer pain and collaborations between pain experts helped advance cancer pain management globally. In 1978, at the first IASP meeting, Kathleen Foleys presentation of the first prevalence study on cancer pain demonstrated the burden of cancer pain, demonstrating that one third of cancer patients in active therapy and two thirds of patients with advanced disease had significant pain (16). In 1982, Jan Stjernswärd, the new head of the World Health Organization (WHO) brought together key international experts including Foley, Robert Twycross, Neil Macdonald and Vittorio Ventafridda, to develop a practical pain regimen for global education and dissemination (17). In 1986, the WHO published its cancer pain relief guidelines, conceptualized as a sequential three-step ladder with administration of non-opioid to weak opioids to strong opioids according to the step (by-the-ladder) on a regular schedule (by-the-clock), (18–20). The WHO pain ladder has been pivotal to cancer pain education and legitimatized the use of morphine and other opioids for cancer pain around the world.

## Cancer pain assessments: From uni- to multidimensional scales

In 1968, Margo McCaffery's simple definition of pain “…its whatever the experiencing patient says it is, existing whenever and wherever the person says it does,” (21) is reflected in the revised IASP pain definition (22). The development of self-reported pain intensity scales started in the 1970's, mainly for use in human analgesic trials (23–25). Beginning in the 1980's, these scales started to permeate into the clinical space, and by the mid-1990's, the American Pain Society (APS) promoted pain assessments in hospitals in a manner similar to vital signs. However, these unidimensional scales were not embraced by hospice specialists until much later, due to fears that the scale would oversimplify the patient's narrative of their suffering. Palliative care clinicians recognized that in addition to the suffering caused by pain, advanced cancer patients experience several concurrent symptoms that affect their pain, and negatively impact quality of life and caregiver burden. In 1991, the Edmonton Symptom Assessment System was developed by Bruera et al. (26) and represents one of the first multi symptom assessment scale in palliative care, and has since been validated by multiple groups, and adopted in both clinical practice and research in many centers worldwide.

## Refinement of opioid use for cancer pain: Pioneering studies

In the UK, Twycross and Saunders at St Christopher's Hospice demonstrated the effectiveness of morphine, diamorphine and methadone in managing cancer pain, and validated the regular prophylactic use of opiods in cancer patients (27–29). The introduction of extended release (ER) opioids helped implement this pioneering concept of regular opioid administrations, as before only small groups of hospice and palliative clinicians were doing this and the emergence of ER preparations helped others such as oncologists provide scheduled opioids to their patients.

Houde and his team at Sloan Kettering pioneered the development and use of methods to assess pain and analgesic therapies for cancer patients (30, 31). His clinical fellow, Foley was tasked to create the first clinical pain research program in the US, and subsequently created the taxonomy of cancer pain (32). Bruera and colleagues demonstrated the feasibility and benefits of continuous opioid administration *via* the subcutaneous route in cancer patients, which offered better pain control and without the discomfort, expense and bolus effect (such as drowsiness or nausea) associated with intermittent injections (33, 34). In 1995, Noémi De Stoutz and Bruera demonstrated the beneficial effects of opioid rotation on side effects and the ability to use lower opioid dosages than those predicted by equianalgesic tables, (35) which is the current practice when clinicians encounter opioid toxicities or in patients who are highly tolerant to opioids (36). Fentanyl and methadone came to be used as second line opioids for refractory pain in the 1990's. The demonstration by Carla Ripamonti and Bruera that the equianalgesic dose ratio between methadone and other opioids is higher than suggested by existing tables and correlation with the total opioid dose before switch (37), and its complex pharmacokinetics (38) necessitates prescribing by experienced clinicians. The introduction of fentanyl as a transdermal system allowed for the delivery of ER opioids in patients unable to swallow, and its more predictable pharmacokinetics as compared to methadone led to its widespread adoption by clinicians caring for cancer patients at the end of life.

## Opioid crisis: The perfect storm of unintended consequences

For the past 2 decades, the U.S. has seen a resurgence of opioid misuse and addiction, just as it did in the late 1800's (2)^2^. Attitudinal shifts toward opioid use began in the 1980's (2). Increased awareness of uncontrolled pain in hospitals, (39) and the teaching at the time that patients rarely develop opioid addiction when prescribed for pain, (40) based on two heavily cited retrospective studies published in the 1980's, (41, 42) that went unchallenged for decades. Compounding this were multisystem regulatory failures that allowed Purdue Pharma to aggressively campaign for opioid use in chronic non-cancer pain patients, and particularly marketed Oxycontin as an ER opioid with low addiction risk, (43, 44) which resulted in it widespread use and contributed to the first wave of the opioid crisis (43). In hospitals, implementation of repeated pain assessments, (45) the Joint Commission's 2001 pain standards tying healthcare quality and patient satisfaction to pain control, (46) and cuts in reimbursement policies for multispecialty care for pain^3^, further led to skyrocketing increase in opioid prescribing, once reserved for severe cancer pain, for the management of non-malignant pain.

The first wave of the opioid crisis started in the early 2000's and was directly attributed to the rise in opioid prescription overdose deaths (see footnote text 2), which resulted in the unprecedented effort to target opioid prescribers, (47) new opioid guidelines from the Centers of Disease Control and Prevention, (48) and the requirement to review prescription drug databases before prescribing or dispensing opioids (49). Since 2011, the U.S. has seen a steady decline in opioid prescribing and although cancer patients are exempt, limited access to opioids has been an unintended consequence of restrictive regulations. These limitations have occurred in the form of opioid shortages, (50) decline in the amount and dosing of opioids to patients with advanced cancers, (51–53) and perceived barriers by cancer patients to receive opioids for their pain (54). The physician's hesitancy to prescribe controlled substances may stem from perceived and real risks associated with regulatory and legal scrutiny (55). Furthermore, the APS declared bankruptcy from litigation costs relating to the allegations of its role in the crisis and closed its operations in 2019 (56).

## Discussion: Lessons learned and current best practices for cancer pain management

We know that pain is just one of the aspects of suffering in cancer patients and other aspects of suffering have major impact on the way patients experience and express their pain. Therefore, efforts to alleviate all those other physical, psychological, social and spiritual factors that contribute to pain expression is pivotal, (57) and inadequate assessment of those is a big barrier to optimal relief (58). Cancer pain should not be managed anymore as an entity but as part of a multidimensional suffering component that needs to be properly assessed and managed ideally by an interdisciplinary care team.

^3^

Opioids, while widely feared due to the association with misuse and addiction are essential due to their effectiveness in managing cancer pain. There is now mounting evidence that patients with cancer who receive opioids might be at a higher risk NMOU than previously believed, (59, 60) with one in five cancer patients at risk (61). Therefore, clinicians need to be mindful of the risks of misuse in cancer patients as well and assume responsibility for risk management even when these drugs are being legitimately prescribed. This involves implementing a practice of adopting universal precautions– to proactively identify and monitor patients at risk for opioid misuse, delineate boundaries for opioid prescribing, and setting realistic expectations for pain relief (62).

## Author contributions

All authors listed have made a substantial, direct, and intellectual contribution to the work and approved it for publication.

## Funding

EB was supported in part by National Institutes of Health Grant Numbers: RO1NR010162-01A1, RO1CA122292-01, and RO1CA124481-01.

## Conflict of interest

The authors declare that the research was conducted in the absence of any commercial or financial relationships that could be construed as a potential conflict of interest.

## Publisher's note

All claims expressed in this article are solely those of the authors and do not necessarily represent those of their affiliated organizations, or those of the publisher, the editors and the reviewers. Any product that may be evaluated in this article, or claim that may be made by its manufacturer, is not guaranteed or endorsed by the publisher.

## Footnotes

^1^ https://www.nhpco.org/hospice-care-overview/history-of-hospice/

^2^ https://www.cdc.gov/drugoverdose/epidemic/index.html

^3^ https://painmed.org/minimum-insurance-benefits-for-patients-with-chronic-pain/
